# The Impact of COVID-19 and Non-COVID-19 Vaccinations in Special Populations

**DOI:** 10.3390/vaccines12010079

**Published:** 2024-01-12

**Authors:** Kay Choong See

**Affiliations:** Division of Respiratory and Critical Care Medicine, Department of Medicine, National University Hospital, Singapore 119074, Singapore; kaychoongsee@nus.edu.sg

Vaccination to prevent human infection is a key driver for reducing morbidity and mortality. However, vaccine hesitancy, defined by the World Health Organization’s Strategic Advisory Group of Experts on Immunization as “the delay in acceptance or refusal of vaccines despite availability of vaccination services” [1], can lead to under-vaccination. One of the reasons for vaccine hesitancy is the lack of confidence, or trust, in the efficacy and safety of vaccination.

To drive and sustain the vaccination effort and broaden the uptake by individuals, clinicians, and policy makers require evidence that demonstrates the efficacy and safety of vaccination not only to the general population, but also to special segments of the population that are prone to vaccine hesitancy. This Special Issue therefore aims to explore the positive and negative impact of COVID-19 and non-COVID-19 vaccination for these special populations. Ten papers have been published in this Special Issue that broadly address four population groups who are prone to vaccine hesitancy and contain information with important clinical and policy implications.

The first population group consists of individuals who are children [2], adolescents [3], and older adults [4]. Osman and colleagues conducted a test-negative matched case–control study among 14,161 children and adolescents aged 12–17 years in Qatar between 1 June and 30 November 2021 and demonstrated that a two-dose primary series of the Pfizer-BioNTech BNT162b2 mRNA COVID-19 vaccine provided a relatively high vaccine efficacy of 79%. A systematic review conducted by Tian and colleagues provides further data from 12 randomized controlled trials that support COVID-19 vaccination in children and adolescents under the age of 18 years by demonstrating high vaccine efficacy, high immunogenicity, and low rates of serious adverse events across various vaccine platforms. Ishak and colleagues focused on adults who are 75 years old and above and reviewed the vaccination recommendations in guidelines for the top 25 non-communicable diseases that are suffered by these older adults. The authors found that the current guidelines do not uniformly provide vaccination recommendations and generally omit information on the benefits and risks of vaccination, highlighting the need for guidelines that provide more comprehensive recommendations to promote vaccination uptake.

The second population group consists of patients with various comorbid conditions that could be perceived to blunt the efficacy of vaccination [5]. Widhani and colleagues performed a systematic review of COVID-19 vaccination in patients with autoimmune diseases. These patients are often immunocompromised from both their disease and from immunosuppressive medications. From the 76 studies included in their review, as expected, compared with healthy controls, patients with autoimmune diseases showed impaired immunogenicity to COVID-19 vaccines. The clinical impact of impaired immunogenicity differed between the vaccine platforms, with a 93% increased risk of breakthrough infections for inactivated vaccines and no increased risk for mRNA or adenovirus vector vaccines. Additionally, they found that a second dose of COVID-19 vaccination increased immunogenicity without elevating the risk of systemic adverse events. Ziemssen and colleagues arrived at similarly encouraging results for a very specific subgroup of 23 patients who received ofatumumab—a human anti-CD20 monoclonal antibody—for relapsing multiple sclerosis and reported good seroconversion rates for the COVID-19 mRNA booster vaccination.

The third population group consists of risk-averse individuals who perceive vaccination-related adverse events to be common or serious [6]. These individuals include those with prior severe drug-, food-, or insect sting-related allergic reactions like anaphylaxis, who were found by Asperti and colleagues to be more anxious when receiving COVID-19 vaccination compared with those with a mild allergy. Such anxiety was lowered by having the vaccination administered in dedicated facilities while supervised by an allergist. Other less acute but serious adverse events might worry some individuals. One of these adverse events is sensorineural hearing loss, which Liew and colleagues studied in their systematic review. The incidence of post-vaccination sensorineural hearing loss was fortunately very low at 0.6–60.77 cases per 100,000 person years for both COVID-19 and non-COVID-19 vaccines, which was comparable to the incidence of all-cause hearing loss, suggesting no excess risk from vaccination. Given the uncertainty about the increased risk of side effects with repeated vaccination, Soegiarto and colleagues studied 75 healthcare workers in Indonesia who received a third dose of heterologous COVID-19 mRNA booster vaccine after a two-dose series of inactivated vaccines. They found that the mRNA vaccination elicited a more robust antibody response compared with a third dose of inactivated vaccine, with minimal systemic side effects. Further evidence of the safety of three vaccine doses comes from an online survey in Saudi Arabia, conducted by Aldali and colleagues. Among 413 participants in the general population, individuals mostly reported mild to moderate side effects lasting less than four days after a three-dose series of various COVID-19 vaccines.

The fourth and final population group consists of individuals who have been previously infected [7]. Qin and colleagues found that nearly 60% of people who have recovered from COVID-19 infection experienced pandemic fatigue, defined by the World Health Organization as the “natural and expected reaction to sustained and unresolved adversity in people’s lives” [8]. As pandemic fatigue has been linked to vaccine hesitancy, this study highlights the need to especially educate and encourage this population segment to receive further vaccination.

In conclusion, the papers in this Special Issue provide good support for vaccination to prevent disease and preserve health. Given the timing of this Special Issue in 2023, which coincided with the COVID-19 pandemic, most papers unsurprisingly involved COVID-19 vaccination. Nonetheless, the contributing authors have provided information that can be generalized to non-COVID-19 vaccination. Addressing the concerns of special populations at both ends of the age spectrum, patients with immunocompromising comorbid conditions, risk-averse individuals, and individuals experiencing pandemic fatigue can then help realize the full value of vaccination to maintain good health, safeguard economic activity, and avoid large-scale societal disruptions like pandemic lockdowns and border closures.
